# Supplementary material for: Local field potentials in the parietal reach region reveal mechanisms of bimanual coordination
Source: Nat Commun. 2021 May 4;12:2514. doi: 10.1038/s41467-021-22701-3 (PMC8096826; doi:10.1038/s41467-021-22701-3)
Supplement: Supplementary file 1 — Supplementary Information [file 41467_2021_22701_MOESM1_ESM.pdf]

Local field potentials in the parietal reach region reveal mechanisms of bimanual coordination

Authors: Eric Mooshagian[1]*, Charles D. Holmes[2], Lawrence H. Snyder[1,2]
Affiliation: [1]Department of Neuroscience, Washington University School of Medicine, St. Louis, MO 63110, USA; [2] Department of Biomedical Engineering, Washington University in St. Louis, St. Louis, MO 63130, USA

SUPPLEMENTARY INFORMATION

Error bars are used throughout to highlight the reproducibility of responses across recording sites. In response to a reviewer query we emphasize that the 'N' used for our error bars and significance calculations is based only on the number of recording sites (or site pairs), not on the product of sites times trials. These error bars therefore provide the astute reader with the means to compute effect size. We use Figure 3a as an example. Standard errors are provided for time course and bar plots. For contramanual reaches (red data), the error bars represent our confidence in the change in power between baseline and the interval from 650 to 1150 ms after target presentation, computed within the band-limited LFP power from 20 to 30 Hz. The standard error is 1.97%, computed across 312 recording sites (see figure legend). The standard deviation is therefore a 34.9% change in power (SEM times square root of [number of recording sites minus 1]). The equivalent values for ipsimanual reaches (green data) are ±2.02% and 35.6%. The standard error of the *difference* is a 1.69% change in power and the standard deviation is 29.8%. (Note that this is somewhat less than the 2.82% and 49.8% that would be predicted for independent data.) The effect size can be quantified as the ratio of the effect size to the standard deviation (Cohen's D). For example, the Cohen's D value for difference in LFP power between for contramanual versus ipsimanual reaching is 21.8% divided by 29.8%, or 0.73 – a large effect size.

Supplementary Table 1

Supplementary Table 1. Overall performance by movement condition during neuronal recording.

Movement Condition	Mean Success Rate (SD)	20th percentile	80th percentile
Saccade	81 (16)	74	93
Left arm	73 (19)	61	89
Right arm	79 (20)	73	94
Bimanual-together	73 (23)	61	91
Bimanual-apart	75 (16)	58	89

Legend: Mean success rates are computed based on the behavioral data collected for each of the reported neurons (n = 113). Percentiles indicate the percentage of individual behavior sets with at least that success rate. SD = standard deviation.

Supplementary Table 2

Supplementary Table 2. Movement duration ± SD, in ms, by movement condition and direction.

Animal	Movement Condition	Movement Direction							
		0°	45°	90°	180°	135°	225°	270°	315°
MkT	Left arm	174 ±14	168 ±14	161 ±20	134 ±14	145 ±17	115 ±11	147 ±20	159 ±24
MkT	Right arm	178 ±30	187 ±17	206 ±28	196 ±21	203 ±22	198 ±32	145 18	131 ±32
MkT	Bimanual-together	176 ±21	201 ±26	193 ±19	179 ±19	192 ±26	170 ±41	144 ±19	157 ±35
MKT	Bimanual-apart	169 ±26	163 ±29	159 ±21	195 ±26	171 ±31	180 ±53	158 19	156 ±28
MkZ	Left arm	263 ±40	278 ±42	258 ±45	176 ±35	204 ±27	222 ±86	177 ±26	222 ±34
MkZ	Right arm	180 ±23	211 ±24	229 ±27	223 ±30	256 ±42	211 ±27	161 ±23	176 ±32
MkZ	Bimanual-together	209 ±32	223 ±31	223 ±33	194 ±27	220 ±36	200 ±48	153 ±20	198 ±40
MkZ	Bimanual-apart	176 ±25	203 ±47	177 ±28	229 ±43	247 ±55	250 ±54	186 ±32	204 ±43

Legend: Movement durations (time between reach onset and endpoint) in milliseconds (ms), with standard deviation. “Movement direction” refers to the endpoint position, coded as the angle in degrees relative to the central fixation. For bimanual-apart movements, this indicates the direction of the right arm moved; the left arm moved toward a target positioned 180° across the fixation point from the target of the right arm.

Supplementary Figure 1

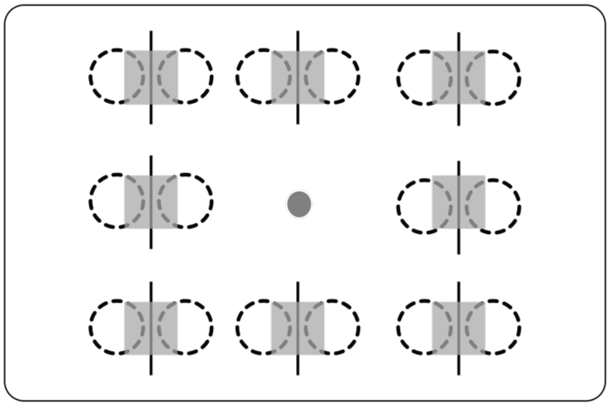

Supplementary Figure 1 (related to Figure 1). Schematic of the touch screen configuration. Targets (gray squares) were arranged in a rectangle around the central fixation point. Touches were detected by capacitive switches mounted behind the Plexiglas touch screen (dashed black circles). To ensure that touches could be correctly attributed to the left or right hand, animals were trained to reach, with the left and right hands, respectively, to the left and right side of a small plexiglass divider mounted to the front of the screen at each touch location (vertical black lines). See text for additional details.

Supplementary Figure 2

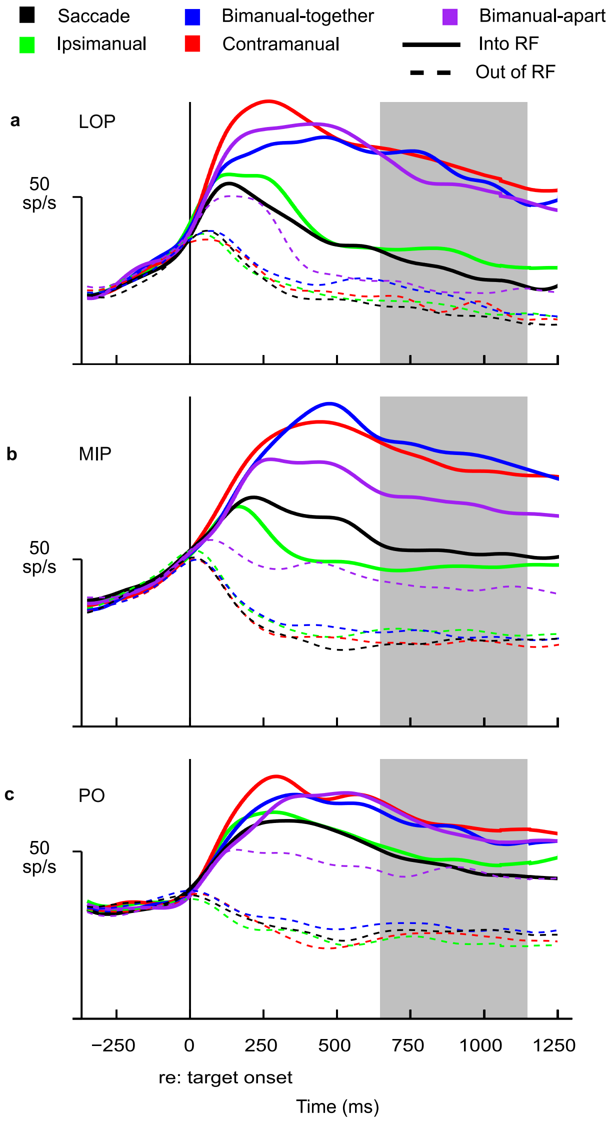

Supplementary Figure 2. PRR population average spiking activity for all ten conditions by anatomical area. a. lateral intraparietal area (LOP). **b.** medial intraparietal area (MIP). **c**. parietal-occipital area (PO). Format same as in Fig. 3. Source data are provided as a Source Data file.

Supplementary Figure 3

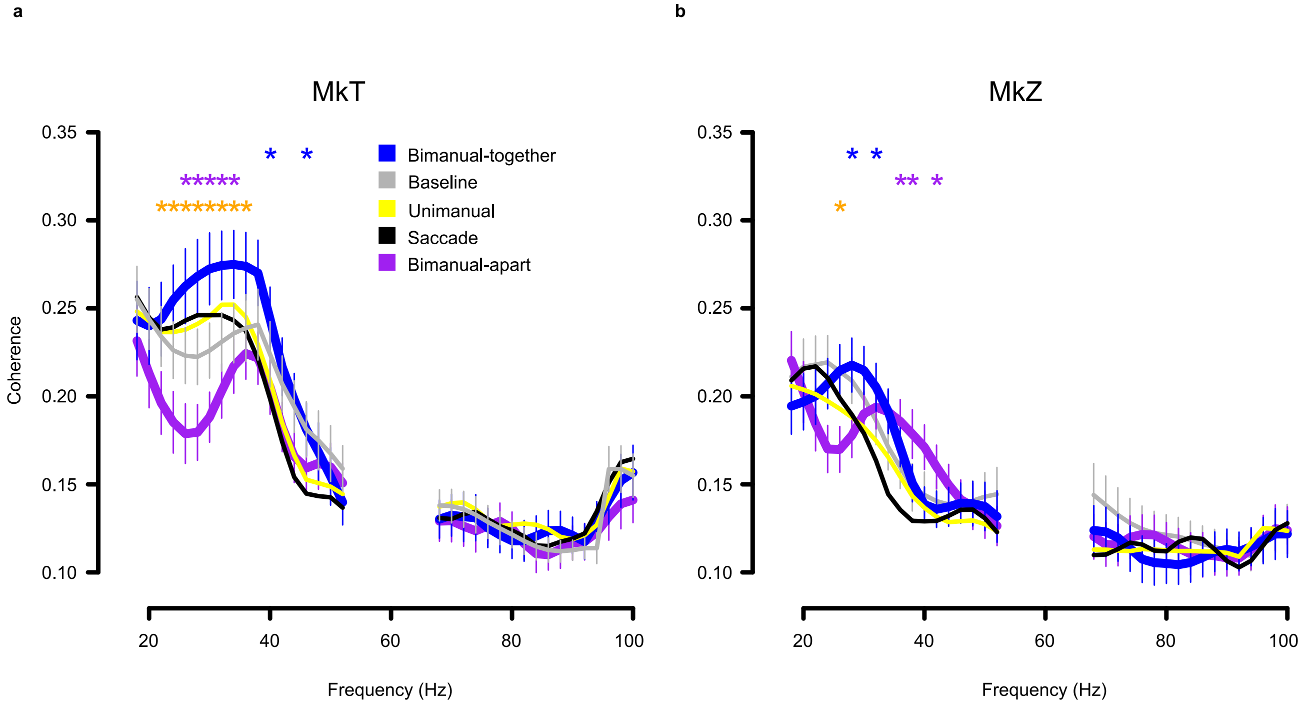

Supplementary Figure 3 (related to Figure 2). Beta-band LFP-LFP coherence by monkey. a. MkT. **b.** MkZ. Beta-band LFP-LFP coherence between PRR in the left and right hemispheres distinguishes bimanual-together and bimanual-apart movements from baseline and from unimanual movements. Coherence in the beta-band (~20-30 Hz) is elevated for bimanual-together movements (blue) and decreased for bimanual-apart movements (purple) compared to unimanual reaches (yellow) or to the baseline period (gray). Coherence during saccade trials (black) resembled that seen during unimanual reach (yellow). Blue and purple asterisks indicate significant differences of bimanual-together and bimanual-apart, respectively, versus unimanual. Gold asterisks denote comparison of bimanual-together versus bimanual-apart (two-sided t-tests, asterisks = $P < 0.05$ after Bonferroni correction for testing at each of the 39 different frequency values plotted in the figure). Data are averaged from n = 55 sites in MkT and n = 64 in MkZ, with coherence measured in the -500 - 0 ms before the cue to move. Format as in Figure 2. Source data are provided as a Source Data file.

Supplementary Figure 4

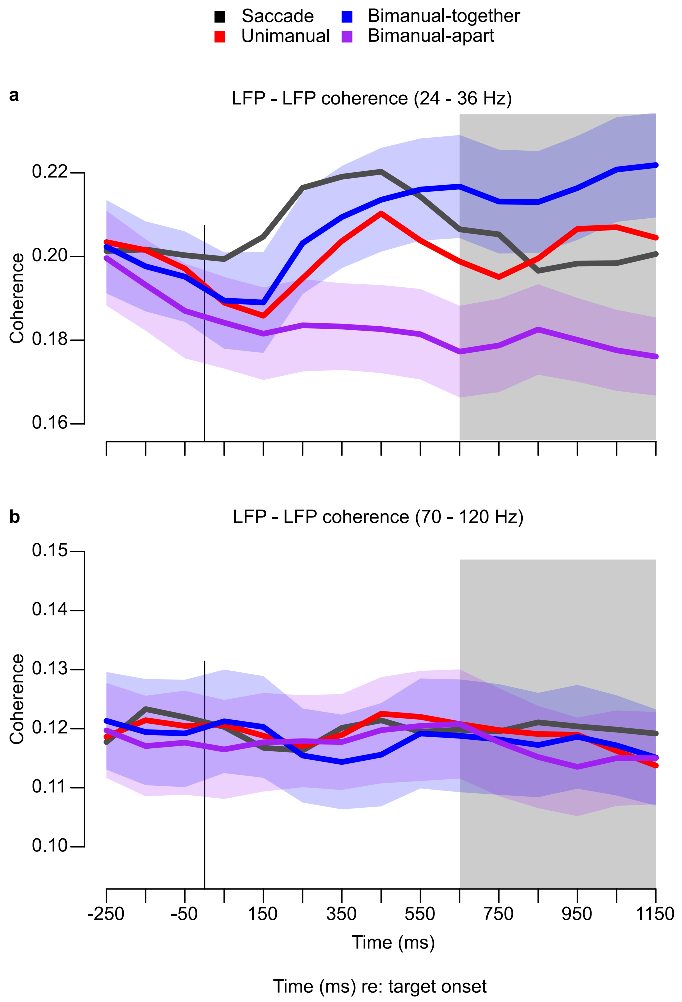

Supplementary Figure 4. LFP-LFP coherence as a function of time through the trial. a. Beta-band coherence (24 – 36 Hz). **b.** Gamma-band coherence (70 – 120 Hz). Trial type first affects beta-band coherence ~250 ms after the reach instruction is delivered, and the effect becomes significant at ~550 ms. This timing is approximate since with more power, that is, with more trials per site or more sites, the effects might become significant earlier. In addition, coherence measures are fundamentally of low temporal resolution, since several cycles are required for an accurate measure [1, their Supplementary Fig. S7]. Gamma-band coherence does not change as a function of time. In this figure, coherence is computed within 400 ms windows, allowing for 10 cycles at 25 Hz, spaced 100 ms apart (n = 113 LFP-LFP pairs). Shading, for bimanual-together and -apart, represents ±1 s.e.m. Source data are provided as a Source Data file.

Supplementary Figure 5

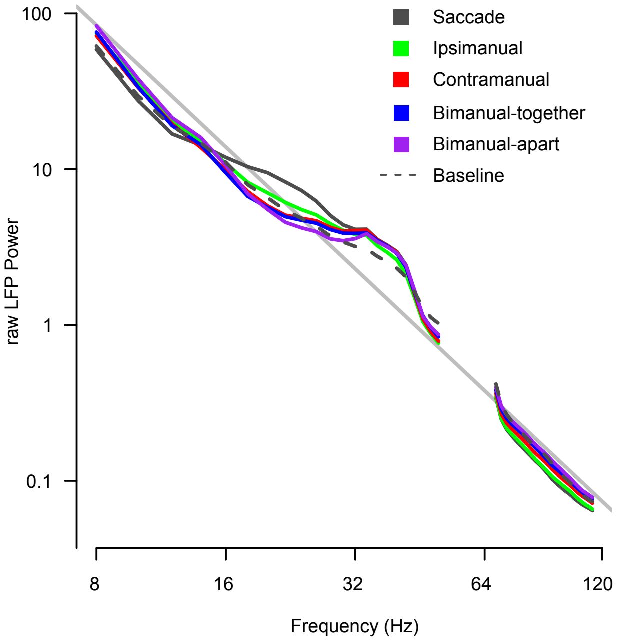

Supplementary Figure 5. Absolute LFP power between 8 and 120 Hz ranges over 3 orders of magnitude. Power is nearly linear with frequency on a log-log plot, meaning that it varies inversely with frequency raised to an exponent ($1/f^N$). The baseline power, measured in a 500 ms interval when the animal is fixating but before the task instruction has appeared (dashed black line) is best fit with an exponent of -2.2 (light gray line; exponents of -2.14 and -2.25 for MkT and MkZ, respectively, not shown). Task-evoked power, measured 650 to 1150 ms after target onset on preferred direction trials (colored solid lines) deviates from the linear fit in the range from 8-40 Hz, with task-specific deviations most prominent from 12-30 Hz. Data are averaged across all 312 recording sites. Source data are provided as a Source Data file.

Supplementary Figure 6

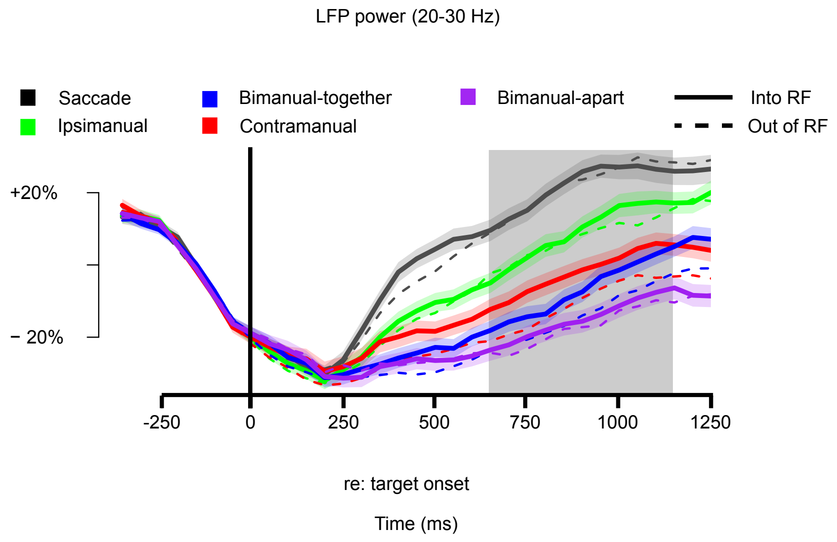

Supplementary Figure 6. **Direction has no systematic effect on beta power.** Preferred directions were assigned based on the preferred direction of a single unit recorded simultaneously and on the same electrode as the LFP (n=113). The beta band LFP power evoked when planning a movement in the preferred direction was very similar to the power evoked when planning a movement in the non-preferred direction (compare solid and dashed lines, respectively). There is no significant effect of direction (preferred versus null) for saccades and for ipsilateral and bimanual-apart reaches (mean power in the interval 650-1150 ms after target appearance, two-sided paired t-tests, n = 113 for each comparison, $P > 0.05$). For contralateral and bimanual-together reaches there is a significant effect of direction that does not survive multiple comparisons (5 comparisons). To increase temporal resolution, power was computed in 200 ms windows every 50 ms. In Figure 2 the traces are smoother because preferred and null traces are averaged together, and because power was computed in 400 ms windows spaced 100 ms apart. Shading shows ±1 s.e.m. for the preferred direction traces; s.e.m. is similar for the null direction traces. Source data are provided as a Source Data file.

Supplementary Figure 7

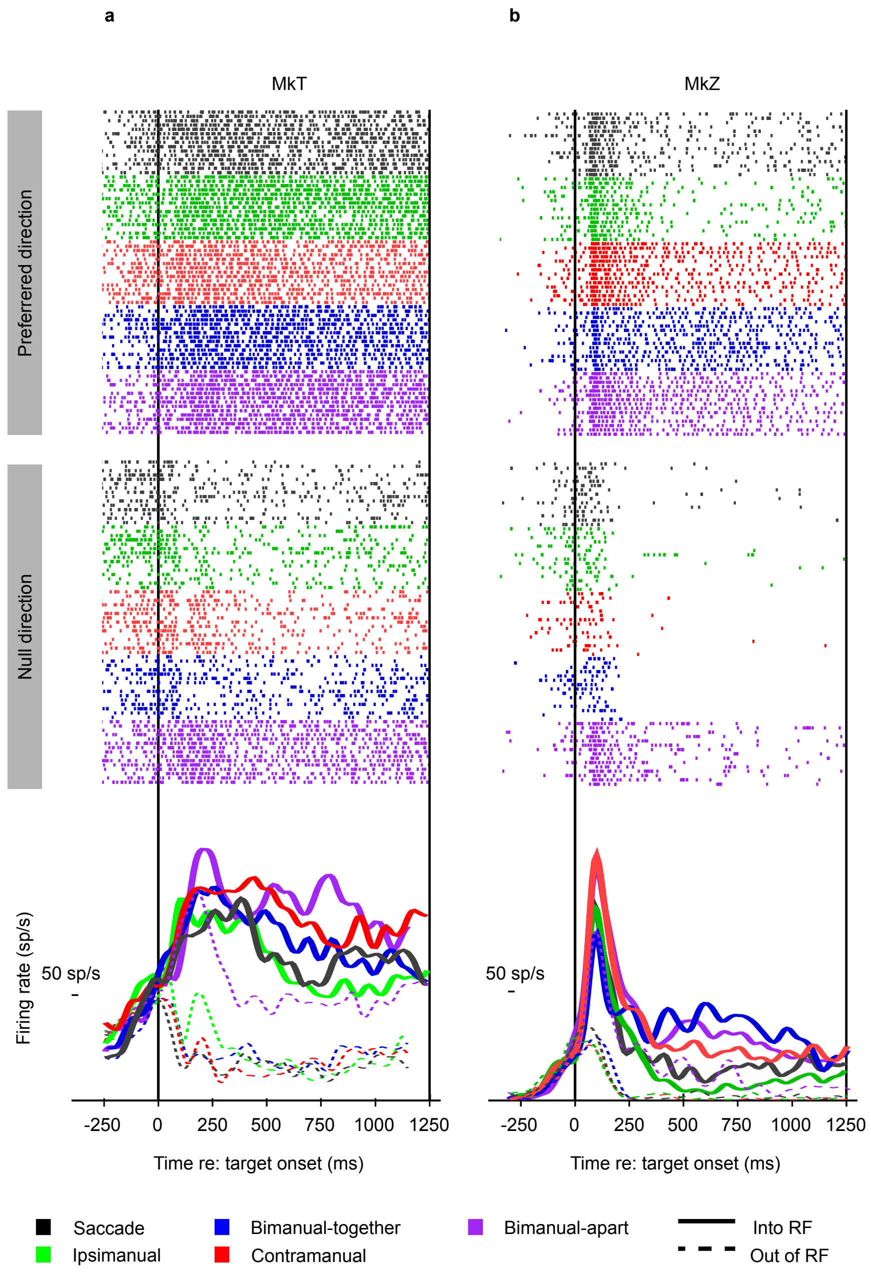

Supplementary Figure 7 (related to Figure 3). Example neurons. Neural signals from typical PRR neurons in MkT (**a**) and MkZ (**b**). Source data are provided as a Source Data file.

Supplementary Figure 8

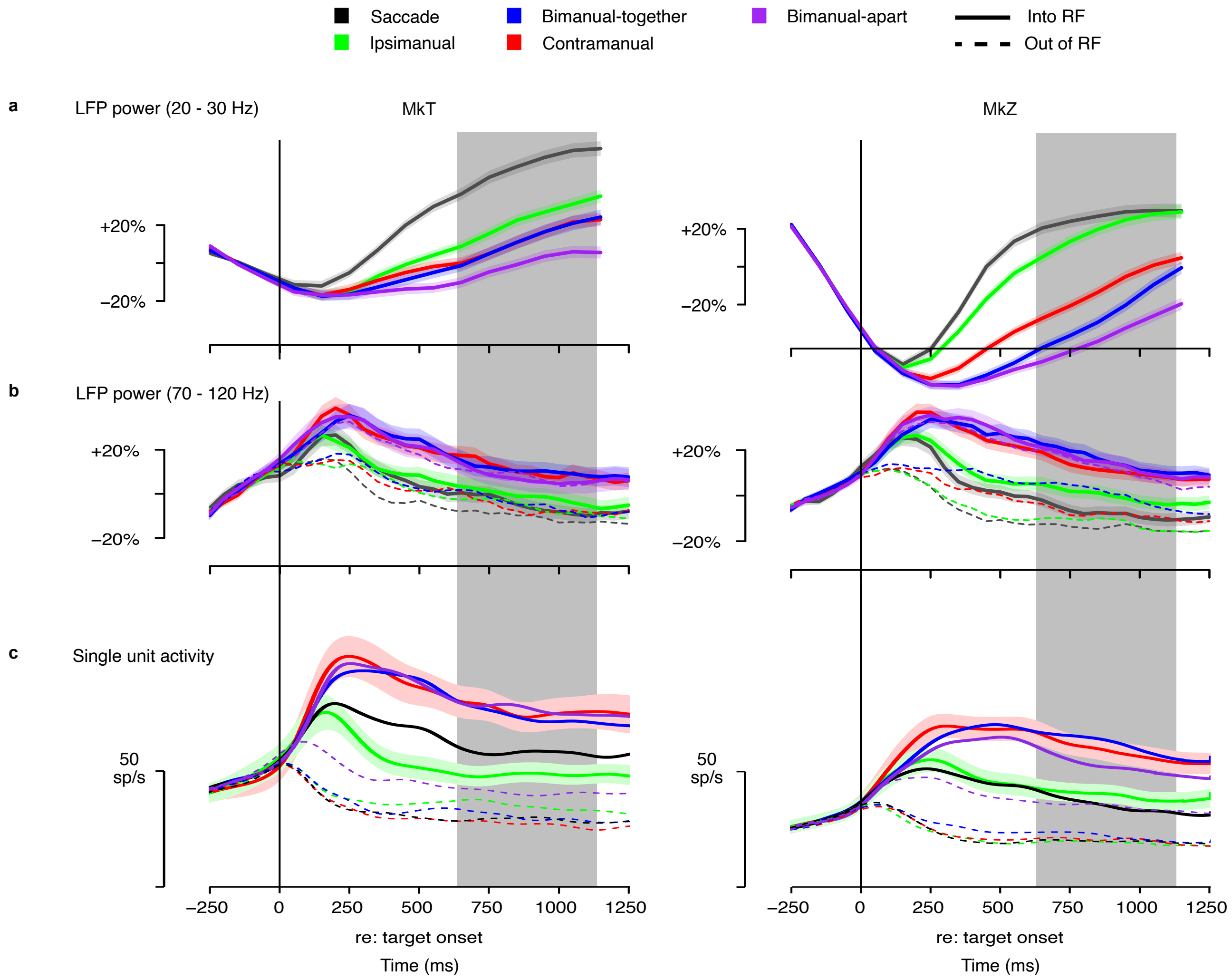

Supplementary Figure 8. (Related to Fig. 3). A comparison of neural activity during 5 movement conditions in each monkey. PRR population average spiking activity for all ten conditions in MkT (left) and MkZ (right). a. Beta-band LFP power (20 – 30 Hz). b. Gamma-band LFP power (70 – 120 Hz). c. Single unit activity. Format same as in Fig. 3. Source data are provided as a Source Data file.

Supplementary Figure 9

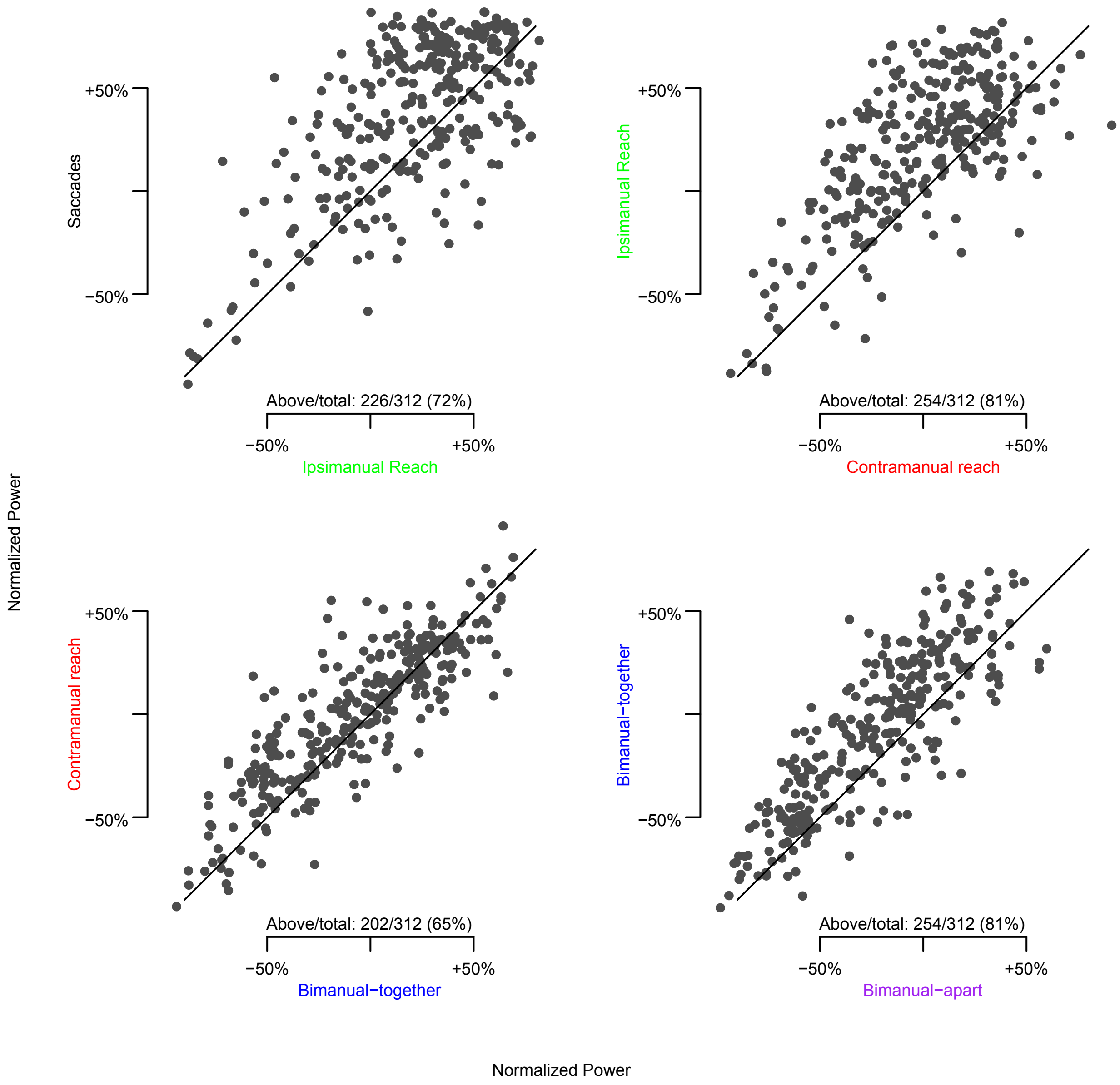

Supplementary Figure 9. Normalized power at 24 Hz in PRR at each individual recording site. The ordering of LFP power at each site generally matches the population average: saccades > ipsimanual reach > contramanual reach > bimanual-together > bimanual-apart. Each point represents a single site during the delay period. Most points (top-left, 75%; top-right, 82%; bottom-left, 67%; bottom-right, 80% in the four plots, which contrast neighboring task types in the population average) fall above the unity line, demonstrating that for most sites, the power for the condition plotted on the y-axis is greater (less suppressed compared to baseline) than the power for the condition plotted on the x-axis. The respective percentages for the data from MkT alone are 92, 68, 47, 81% and for MkZ are 63, 92, 81, 79%. Data are for 650 to 1150 ms after target presentation; other intervals show similar effects. Source data are provided as a Source Data file.

Supplementary Figure 10

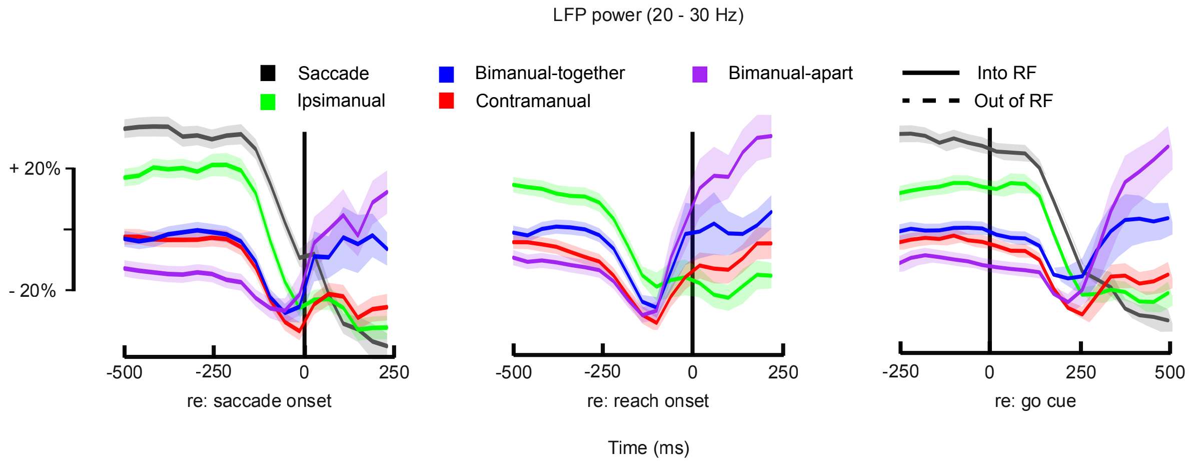

Supplementary Figure 10. A comparison of beta power during the 5 tasks. The data are aligned on saccade onset (left) and reach onset (center) and go cue (right). Format otherwise identical to Figure 2. Beta power drops steeply starting ~125 ms after the go cue (last column), reaching a nadir 50 ms prior to a reach and then increasing steeply on trials with contramanual reaches. Power remains flat after the onset of an ipsimanual reach (middle column) and continues to fall for at least 250 ms after a saccade-alone (no reach) trial (first column). Source data are provided as a Source Data file.

Supplementary Figure 11

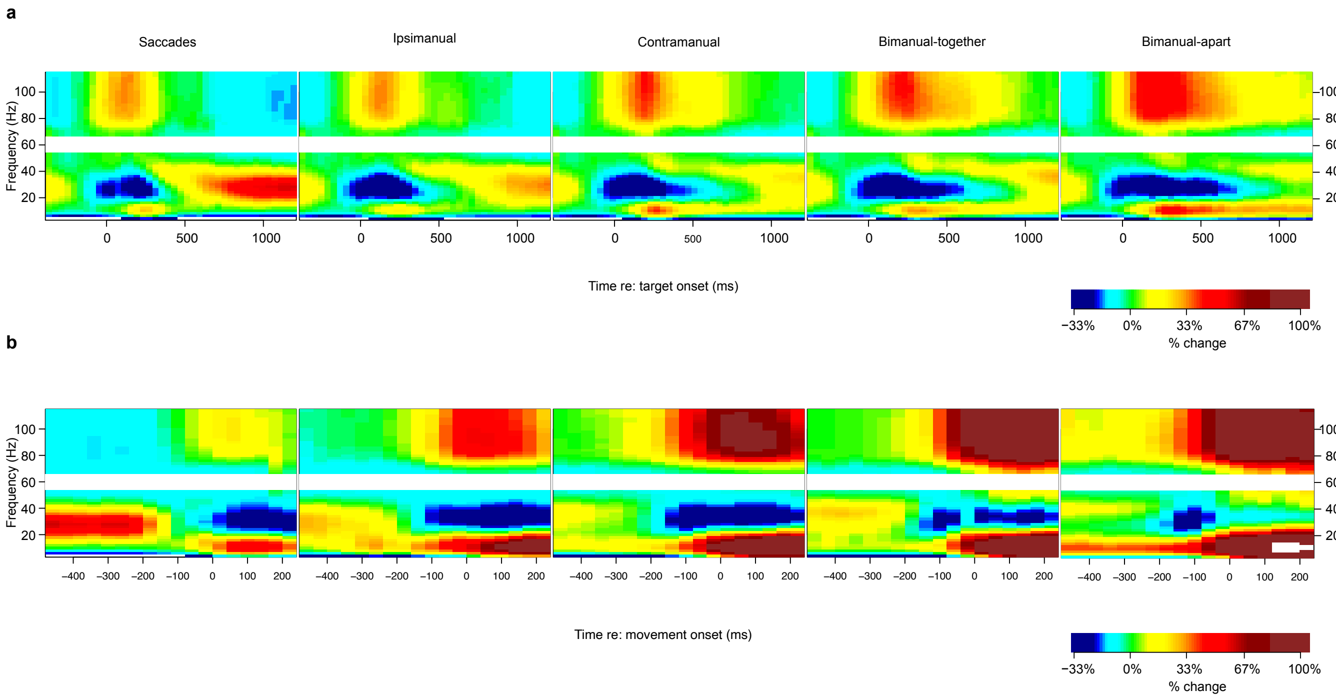

Supplementary Figure 11. **Population spectrograms of the LFP. a**. LFP power (0 – 100 Hz) as a function of time for each movement type during the delay period aligned to target onset. **b**. Same as **a** except data are aligned to movement onset (saccade onset for the first column, reach onset for the remaining columns). Color code indicates the LFP power relative to the baseline period (500 ms prior to target onset). In **b**, white corresponds to power greater than 150%. Source data are provided as a Source Data file.

Supplementary Figure 12

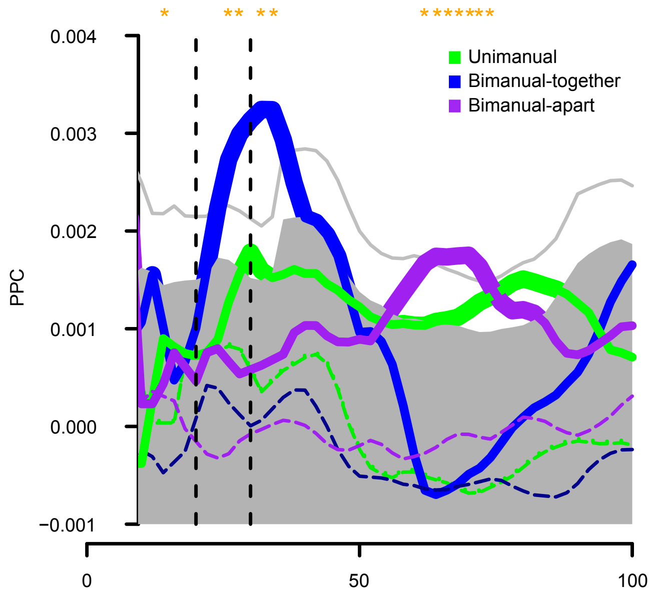

Supplementary Figure 12 (related to Figure 5). Spike-LFP coherence computed using pairwise phase consistency (PPC). PPC is designed to avoid the confounds of too few spikes that influence spike-LFP coherence measurements. Therefore, here we include all cells with at least 100 spikes in the last 600 ms before the go cue (n = 80 spike-field pairs). Format same as in Fig. 5c. Source data are provided as a Source Data file.

Model Details

We asked if the LFP power modulation during the planning period is a linear sum of the average modulation of firing rates in the preferred and null directions for the ipsimanual and contramanual arms. The population data were fit to a two-term algebraic model, according to Eq. (9)

$$\text{Mean LFP power modulation} = -\left(b1 * \left(\frac{Mod_p + Mod_n}{2}\right)_C + b2 * \left(\frac{Mod_P + Mod_n}{2}\right)_I\right), \quad (9)$$

where *Mod* is the mean modulation of the population average firing rate, the subscripts *p* and *n* indicate the preferred and null directions, respectively, the subscripts *C* and *I* are the contramanual and ipsimanual arm movements, respectively, and b1 and b2 are the relative weights for the firing rates for the inputs from the contramanual and ipsimanual hemispheres, respectively, and b1 and b2 sum to 1. Finally, the value is negated to reflect the sign reversal between spikes and LFP power. We used the mean preparatory firing rate from 650 to 1150 ms after the target onset as input values (**Fig. 3c)**.

Firing rates fell into one of three levels. A null direction saccade or null direction reach with either arm to a single target was associated with a firing rate of ~12 sp/s. Baseline firing (250 ms prior to the target instruction) for all conditions was also ~12 sp/s, so the net modulation was ~0 sp/s. A preferred direction saccade, preferred direction ipsimanual reach, or a bimanual-apart reach with the contralateral arm moving in the null direction all resulted in a rate of ~24 sp/s, or a modulation of ~12 sp/s. A unimanual reach with the contramanual arm, both arms together in the preferred direction, or a bimanual-apart reach with the contralateral arm moving in the preferred direction all resulted in a rate of ~36 sp/s, or a modulation of ~24 sp/s.

Columns 2 and 3 of **Supplementary Table 3** show the modulation in spiking rates in ipsilateral PRR associated with preferred and null direction movements, respectively. Column 4 shows the mean of columns 2 and 3. Columns 5-7 show spiking rates in contralateral PRR. Columns 8-11 show the model output based on weighted sums of local and distal spikes. Given the inverse relationship between LFP power and spikes (**Fig. 3**), we assume that both ipsilateral and contralateral spiking inputs *suppress* beta power. If beta frequency LFP is driven only by local spikes, then the power levels in **Fig. 3a** should be suppressed, or ordered inversely, from the values in column 8 (labelled "100:0"). The observed LFP data do not match these predictions. In contrast, columns 9 and 10 assume that beta frequency LFP is mostly driven by local spikes plus small contributions (5% or 20%) from contralateral PRR. The predictions from both of these models (5 and 20%) match the observed data, with a unique power level for each movement type. The relative order of power is captured but not their specific values (see Discussion). In fact, the predicted order is independent of the input ratio, so long as both hemispheres contribute, and the local hemisphere provides more than half of the input. The order is also independent of the values assigned to each level of neuronal modulation (e.g., 0, 12 and 24 spike/s) as long as these levels are strictly monotonically increasing. The order does not depend on the baseline rate. Finally, although spike rates and LFP power levels change over time, their ordering remains consistent over the entire preparatory period, and so our results do not depend on the particular time interval from which measurements were taken.

We did not attempt to fit the data to an optimal local to distal input ratio. One could do so, but there are numerous factors that would need to be considered in such an attempt. For example, there is no reason to assume that the degree of suppression will be linear with the rate of the spiking input. In fact, since suppression is bounded in one direction (power cannot drop below zero), there may be a compressive relationship between spike rate and suppression as is seen with shunting inhibition [2], such that progressively greater spike rates exert progressively less of a suppressive effect on LFP power. The choice of this relationship, which could be 1/x, logarithmic or one of many other functions, is likely

to have an outsized influence on the fit. In addition, there is a lag between when a spike is produced and when it affects the LFP, and this lag may be different for spikes from the local versus distal hemispheres. Accounting for these additional factors in the model (ratio of local to distal input, shape of the compressive function, lag times, etc.) would improve the fit, but the fact that those variables are poorly constrained would limit the value of such a model.

Supplementary Table 3

Supplementary Table 3. Predictions of LFP power by task type based on local and distal cell firing rates

	Contribution, re: LFP site on right, from:						Net input shaping the LFP			
	Right (local) hemisphere			Left (distal) hemisphere			Ratio, local to distal input:			
Movement Condition	Preferred	Null	Mean	Preferred	Null	Mean	100:0	95:5	80:20	20:80
Saccade	12	0	6	12	0	6	6	6	6	6
Right arm	12	0	6	24	0	12	6	6.3	7.2	9.6
Left arm	24	0	12	12	0	6	12	11.7	10.8	8.4
Bimanual together	24	0	12	24	0	12	12	12	12	12
Bimanual apart	24	12	18	12	24	18	18	18	18	18

Legend: Predictions of LFP power levels across movement condition types. LFP power is recorded from the right hemisphere and is driven by combinations of local (ipsilateral or right hemisphere) and distal (contralateral or left hemisphere) single unit activity. Columns 2-4 model potential LFP inputs originating from local (ipsilateral) single units. Columns 5-7 show similar computations for inputs originating from the distal (contralateral) hemisphere. As an example, consider a preferred direction reach with the right arm. The right arm is ipsilateral to the right hemisphere, and so, based on the data of **Fig. 3c**, we expect 12 sp/s of modulation in the local units (column 2). The right arm is contralateral to the left hemisphere, and so we expect 24 sp/s of modulation in the distal units (column 5). Since the null direction reach drives no modulation in either hemisphere (columns 3 and 6), the resulting mean activities in each hemisphere are 6 and 12 sp/s, respectively (columns 4 and 7). Based on the mean values from columns 4 and 7, we model the effects of different combinations of local and distal input on the beta frequency LFP: 100% local input, 95% local, 80% local and 20% local for columns 8 through 11, respectively. Values are in spikes per second (sp/s) of input, but we assume that these represent the amount of suppression imposed, so that smaller values should result in higher beta power. The important finding from this model is that a mixture of ipsilateral and contralateral input replicates the observed LFP power levels across trial types. See text for details. Preferred = movements into the RF; Null = movements out of the RF; Mean = (Preferred + Null)/2.

Supplementary Figure 13

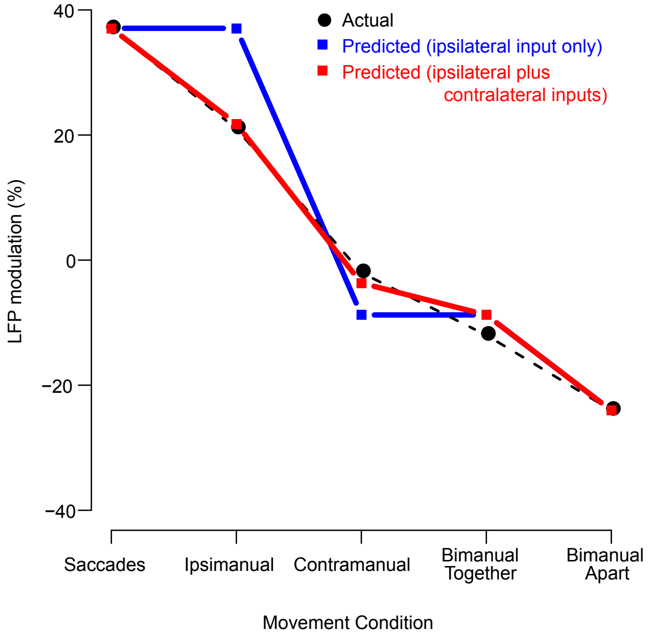

Supplementary Figure 13. Observed and predicted LFP power levels for each movement condition. Each movement condition can be clearly distinguished in the observed LFP power data (black). A weighted sum of ipsilateral and contralateral spikes replicates the observed LFP power levels across movement condition (red). Predicted LFP power recapitulates the spike response when based on only local spikes (blue). Predicted values are scaled to span the same range of the observed values, producing exact fits for both models to the saccade and bimanual conditions. The only other degree of freedom in the model is the relative amounts of local to distal input (80% and 20%, respectively). See Supplementary Information for details. Source data are provided as a Source Data file.

References

1. Li, J. M., Bentley, W. J. & Snyder, L. H. Functional connectivity arises from a slow rhythmic mechanism. *Proceedings of the National Academy of Sciences* **112**, E2527-35 (2015).

2. Prescott, S. A. & Koninck, Y. D. Gain control of firing rate by shunting inhibition: Roles of synaptic noise and dendritic saturation. *Proceedings of the National Academy of Sciences of the United States of America* **100**, 2076–2081 (2003).
